# Interactions between dietary patterns and genetic factors in relation to incident dementia among 70-year-olds

**DOI:** 10.1007/s00394-021-02688-9

**Published:** 2021-10-10

**Authors:** Jessica Samuelsson, Jenna Najar, Ola Wallengren, Silke Kern, Hanna Wetterberg, Madeleine Mellqvist Fässberg, Henrik Zetterberg, Kaj Blennow, Lauren Lissner, Elisabet Rothenberg, Ingmar Skoog, Anna Zettergren

**Affiliations:** 1 Neuropsychiatric Epidemiology Unit, Department of Psychiatry and Neurochemistry, Institute of Neuroscience and Physiology, Sahlgrenska Academy, Centre for Ageing and Health (AGECAP) at the University of Gothenburg, Wallinsgatan 6, 431 41 Mölndal, Sweden; 2 Region Västra Götaland, Sahlgrenska University Hospital, Psychiatry, Cognition and Old Age Psychiatry Clinic, Gothenburg, Sweden; 3 Clinical Nutrition Unit, Sahlgrenska University Hospital, Gothenburg, Sweden; 4 Clinical Neurochemistry Laboratory, Sahlgrenska University Hospital, Mölndal, Sweden; 5 Department of Psychiatry and Neurochemistry, Institute of Neuroscience and Physiology, Sahlgrenska Academy at the University of Gothenburg, Gothenburg, Sweden; 6 UK Dementia Research Institute at UCL, London, UK; 7 Department of Neurodegenerative Disease, UCL Institute of Neurology, London, UK; 8 School of Public Health and Community Medicine, Institute of Medicine, Sahlgrenska Academy, University of Gothenburg, Gothenburg, Sweden; 9 Faculty of Health Sciences, Kristianstad University, Kristianstad, Sweden

**Keywords:** Dietary pattern, Nutrition, Dementia, Apolipoprotein E genotype, Polygenic risk score

## Abstract

**Purpose:**

To investigate potential interactions between dietary patterns and genetic factors modulating risk for Alzheimer’s disease (AD) in relation to incident dementia.

**Methods:**

Data were derived from the population-based Gothenburg H70 Birth Cohort Studies in Sweden, including 602 dementia-free 70-year-olds (examined 1992–93, or 2000–02; 64% women) followed for incident dementia until 2016. Two factors from a reduced rank regression analysis were translated into dietary patterns, one healthy (e.g., vegetables, fruit, and fish) and one western (e.g., red meat, refined cereals, and full-fat dairy products). Genetic risk was determined by *APOE ε4* status and non-*APOE* AD-polygenic risk scores (AD-PRSs). Gene–diet interactions in relation to incident dementia were analysed with Cox regression models. The interaction *p* value threshold was < 0.1.

**Results:**

There were interactions between the dietary patterns and *APOE ε4* status in relation to incident dementia (interaction *p* value threshold of < 0.1), while no evidence of interactions were found between the dietary patterns and the AD-PRSs. Those with higher adherence to a healthy dietary pattern had a reduced risk of dementia among *ε4* non-carriers (HR: 0.77; 95% CI: 0.61; 0.98), but not among *ε4* carriers (HR: 0.86; CI: 0.63; 1.18). Those with a higher adherence to the western dietary pattern had an increased risk of dementia among *ε4* carriers (HR: 1.37; 95% CI: 1.05; 1.78), while no association was observed among *ε4* non-carriers (HR: 0.99; CI: 0.81; 1.21).

**Conclusions:**

The results of this study suggest that there is an interplay between dietary patterns and *APOE* ε*4* status in relation to incident dementia.

**Supplementary Information:**

The online version contains supplementary material available at 10.1007/s00394-021-02688-9.

## Introduction

Genetic and lifestyle factors influence the risk of developing dementia [1, 2]. Diet is one of the modifiable lifestyle factors thought to affect risk [3, 4], but whether there is an interplay with genetic risk factors is unclear [5, 6]. Several nutrients and foods have been linked with the risk of developing dementia [7]. However, since foods are eaten in combination and contain multiple nutrients that might influence risk, there has been a shift from investigating single nutrients and foods towards investigating the impact of dietary patterns [8]. Healthier Mediterranean-style dietary patterns (MeDi), rich in foods such as wholegrain products, vegetables, pulses, fruits and berries, nuts and seeds, fish and seafood have been associated with reduced risk of dementia [9, 10], while western style dietary patterns with a higher content of foods such as red and processed meat, full-fat dairy products, refined cereal products, sweets and high-sugar drinks have been associated with increased risk [7, 8, 11, 12]. However, people often eat a combination of healthy and unhealthy diets, and the cumulative effects of various nutrients and foods may affect risk differently depending on the combinations [13]. Studying the effect of different dietary patterns could, therefore, be useful to increase the understanding of food and nutrient combinations that may prevent or delay the onset of dementia.

The apolipoprotein E (*APOE*) gene is the strongest genetic factor modulating risk for AD and dementia [14]. This gene has three common alleles, the protective allele *APOE ε2*, the neutral allele *APOE ε3*, and the risk allele *APOE ε4* [14]. Through large genome-wide association studies (GWASs), additional AD-risk-modifying genetic variants have been identified [15]. These genetic variants have lower effect sizes and are often combined into polygenic risk scores (PRSs) [16]. Population-based studies have shown that PRSs for AD are associated with AD and all-cause dementia [14, 17, 18], with disease progression [19], and with AD-pathology [17, 20–22].

It has been suggested that risk reducing effects from foods may differ depending on an individual’s genetic risk profile [23–25]. However, previous studies investigating interactions between dietary patterns and genetic risk factors in relation to cognitive function and dementia are limited (especially when it comes to non-*APOE* PRSs) and results are inconclusive, showing either no gene-diet interactions or risk reducing effects among either carriers or non-carriers of genetic risk factors [23, 25–27]. Results from studies investigating interactions between genetic risk factors (usually *APOE*) and single foods or nutrients showed similar contradictive results [28–31].

The aim of this study was to investigate potential interactions between dietary patterns and genetic factors modulating risk for AD (i.e., *APOE ε4* status and non-*APOE* AD-PRSs) in relation to incident dementia among 70-year-olds.

## Materials and methods

**Fig. 1:**
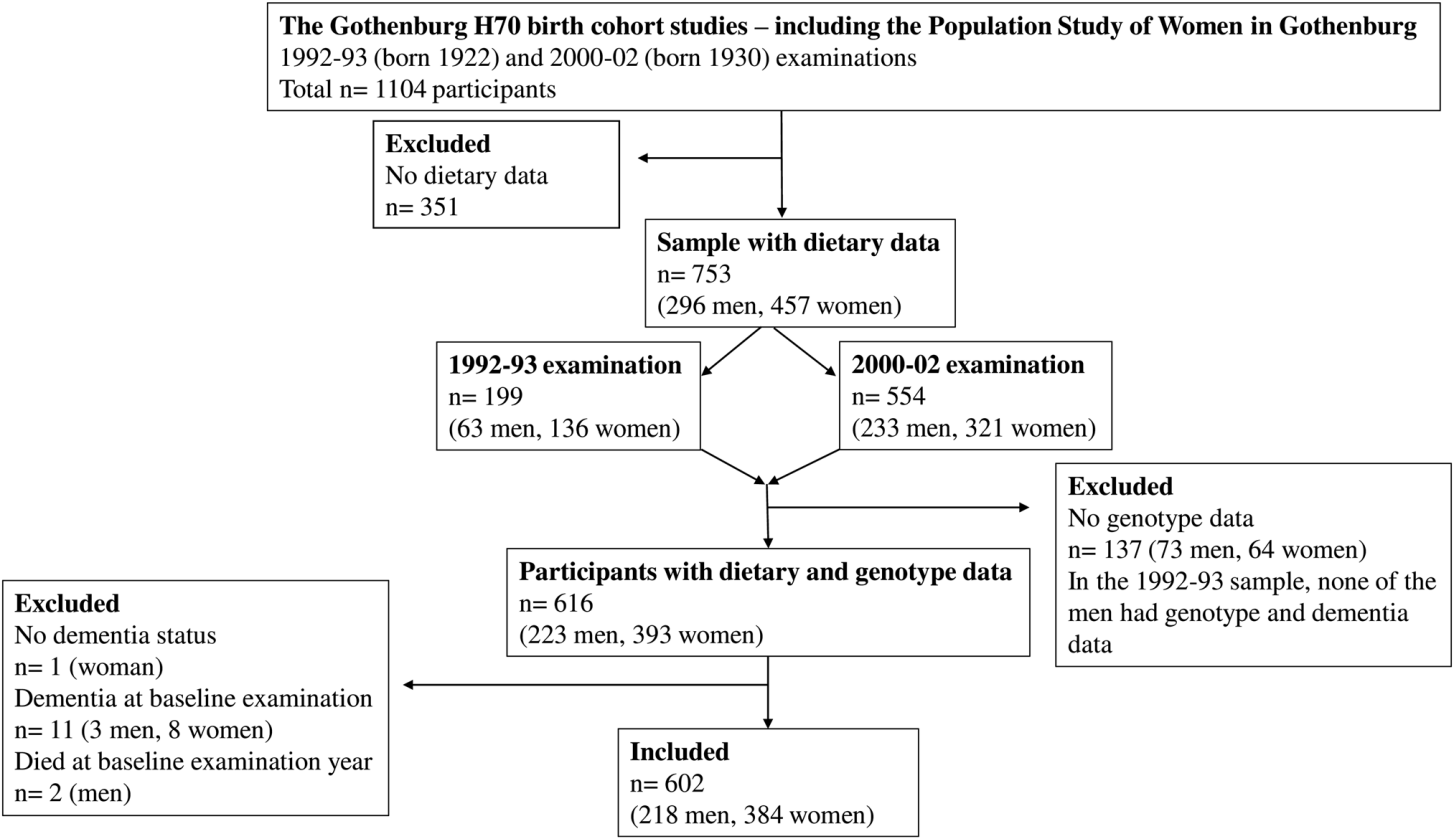
Sample flowchart

Data were derived from the ongoing population-based Gothenburg H70 birth cohort studies that started in 1971 [32], including the Population Study of Women in Gothenburg that started in 1968 [33]. Adults aged 70 years and living in Gothenburg at the time of selection were systematically selected based on birth dates. The Gothenburg H70 birth cohort studies include a wide range of examinations such as genetic, somatic, cognitive, psychiatric, and dietary examinations [34]. This study includes participants born 1922 and 1930, who were examined 1992–93 (born 1922) or 2000–02 (born 1930) and re-examined in 2000, 2005, 2009 and 2015–16. Dementia diagnosis for participants that were lost to follow-up between 2009 and 2015–16 were based on information from the Swedish inpatient registry until 2012 (there were no registry data on dementia diagnosis between 2012 and 2016 available). Information on deaths during follow-up was obtained from the Swedish population registry until 2016. There were 500 (66% response rate) systematically selected 70-year-olds that participated in the 1992–93 examination, and the dietary examination was performed on a subsample of these participants (*n* = 199). There were 604 (71% response rate) systematically selected 70-year-olds that participated in the 2000–02 examination, and all of them were invited to take part of the dietary examination (*n* = 554 participated). Out of the 1104 participants with baseline examinations in either 1992–93 (born 1922) or 2000–02 (born 1930), there were a total of 753 (296 men and 457 women) with dietary data. Blood sampling for genetic analyses was performed in 2000–02. Out of the 753 participants with dietary data, there were 616 (393 women and 223 men) participants with genotype data and 615 (392 women and 223 men) with dementia data. The final sample comprised 602 (218 men and 384 women) participants who were free from dementia at baseline examinations, see sample flowchart in Fig. 1.

### Dietary examination procedure

Information on dietary intake was obtained at baseline examinations (1992–93 for those born 1922, and 2000–02 for those born 1930) with the diet history method (DH) [35, 36]. The DH used in this study was a semi-structured face-to-face interview, estimating food intake during the preceding 3 months. Trained registered dietitians performed the interviews at the participants own home or at the clinic. The protocols for the interviews consist of a meal-pattern interview, accompanied by a food list with questions on usual frequencies and portion sizes of foods. Pictures of foods from the Swedish National Food Agency (NFA) were used during the interviews to estimate individual portion sizes. Dietary intake was registered in grams of food items usually consumed per day/week/month in the NFA’s nutrient database (PC-kost) in 2000–02. The DH method has been validated and described in detail previously [35–37].

### Dietary variables and construction of dietary patterns

Mean daily energy, nutrient and food intake were calculated based on results from the DH interview. Reported food intake was placed into 30 food groups based on similarity of nutritional properties and biological classifications [38] (Supplementary Table 1) and transformed with Box–Cox transformation to normalize the distribution. The Box–Cox transformation is a family of power transform functions that are used to stabilize variance and make a dataset approach normal distribution. Dietary patterns were derived with reduced rank regression analysis (RRR) on the total dietary sample (*n* = 753). The RRR analysis was performed in SAS version 9.4 to reduce the data into factors representing dietary patterns with the 30 food groups as predictor variables [39]. The SAS code used for the RRR analysis has been described in detail previously [39]. The response variables in the RRR analysis were estimated vitamin E, C, folate, fibre, polyunsaturated fatty acids, saturated fatty acids, and alcohol intake (from the DH interview). These nutrients were selected as response variables based on potential associations with dementia risk. Vitamin E, C, folate, fibre, and polyunsaturated fatty acids have been associated with reduced risk of dementia, whereas saturated fatty acids and higher alcohol intake with increased risk [3, 40–46]. The aim of RRR is to explain as much variation of the response variables as possible, by simultaneously reducing the dimensionality of the predictor variables [47, 48]. We chose the RRR approach since it allows us to explore dietary patterns in this population while still considering a priori knowledge about nutrients that have been associated with incident dementia. Data on nutritional supplements were not available.

Two out of five factors from the RRR analysis could be translated into dietary patterns based on factor loading thresholds ≤ − 0.20 and ≥ 0.20 (Table 1). The factor loading thresholds were chosen based on previous studies where dietary patterns have been derived with RRR [49–51]. Factor loadings in between − 0.2 and 0.2 (close to 0) can be considered weak and do not explain the variation in the response variables well. The additional three factors were not examined further since they did not add to explaining the variation in the specified response variables (food group factor loadings were in between ≤ − 0.20 and ≥ 0.20 for most food groups). Factor 1 loaded high on foods found in healthier dietary patterns (e.g., MeDi) such as vegetables, pulses, fruits, berries, fibre-rich bread, fish and seafood, and a higher adherence correlated strongest (of the two dietary patterns) with higher intakes of vitamin C, E, folate, fibre, and polyunsaturated fatty acids (Table 2). Factor 1 was, therefore, labelled the “healthy” dietary pattern (Table 1). Factor 2 loaded high on foods found in less healthy western style dietary patterns such as read meat and processed red meat, refined bread, full-fat dairy products and alcohol and low on vegetables, pulses, fruits and berries, and a higher adherence correlated strongest (of the two dietary patterns) with higher intakes of saturated fatty acids, alcohol, and with lower intakes of vitamin C, folate, and fibre (Table 2). Factor 2 was, therefore, labelled the “western” dietary pattern (Table 1).

**Table 1:** Food item factor loadings for the “healthy” and the “western” dietary patterns derived by reduced rank regression analysis

Dietary patterns	Healthy (factor 1)	Western (factor 2)
Variation in responses (%)	33.3	14.9
Variation in predictors (%)	6.5	6.2
Fish and seafood	0.29*	0.11
Meat and processed meat	0.29*	0.35*
Poultry	0.17	− 0.03
Eggs	0.31*	0.18
Potatoes	0.30*	0.20*
Vegetables, pulses, nuts and seeds	0.53*	− 0.50*
Fruits and berries	0.42*	− 0.57*
Keyhole^a^ milk products	0.05	− 0.29*
Non-Keyhole^a^ milk products	0.08	0.21*
Cream and crème fraiche	0.16	0.26*
Cheese	0.34*	0.24*
Fast food and savoury bakery	0.13	0.08
Pasta, rice, and food grain	0.15	− 0.13
Bread refined ≤ 5% fibre content	0.10	0.31*
Bread fibre-rich > 5% fibre content	0.39*	− 0.13
Cereals	0.27*	− 0.15
Sweet bakery	0.22*	0.22*
Desserts	0.25*	0.02
Sweet condiments	0.29*	0.16
Sweets, candy, and chocolate	0.11	0.14
Soups	0.18	− 0.13
Sauces, dressings, and condiment	0.18	0.05
Margarine	0.36*	0.50*
Butter	0.04	0.19
Vegetable oil	0.28*	− 0.08
Juice	0.28*	− 0.17
Coffee	0.04	0.08
Tea	0.18	− 0.10
Soda	0.13	0.15
Alcoholic beverages	0.28*	0.43*

The content of each food group can be found in supplementary file 1. The dietary patterns were derived with reduced rank regression analysis (RRR) on the total dietary sample (*n* = 753), producing an individual score for each participant

*Two factors from the RRR were translated into dietary patterns based on factor loading thresholds ≤ − 0.20 and ≥ 0.20

^a^Keyhole is the Swedish National Food Agency-labelling scheme, which guides healthy food choices. For milk and yogurt to meet the criteria for the Keyhole, fat content must be limited to a maximum of 0.7%, and for flavoured products there is an additional limit for sugars with a maximum of 9%

**Table 2:** Correlations between extracted dietary pattern scores and response variables from the reduced rank regression analysis

	Healthy dietary pattern (factor 1)	Western dietary pattern (factor 2)
Response variables		
Vitamin E (mg/day)	0.67	0.13
Vitamin C (mg/day)	0.50	− 0.41
Folate (µg/day)	0.73	− 0.26
Saturated fatty acids (g/day)	0.45	0.60
Polyunsaturated fatty acids (g/day)	0.61	0.36
Fibre (g/day)	0.70	− 0.39
Alcohol (g/day)	0.21	0.40

*p* values for all correlations were < 0.0001. The dietary patterns were derived with reduced rank regression analysis on the total dietary sample (*n* = 753), producing an individual score for each participant

### Dementia diagnosis

Dementia was diagnosed at the examinations (1992–93, 2000–02, 2005, 2009, 2015–16) following the Diagnostic and Statistical Manual of Mental Disorders, Third edition, Revised criteria [52], using information from comprehensive neuropsychiatric examinations, a battery of neuropsychological tests and information from close informants, described in detail previously [34]. The same method was used at all examinations for comparability over time. Age of dementia onset was based on information from close informants, the examinations, or the Swedish inpatient register. If no information of age at dementia onset could be obtained, the midpoint between examinations (last examination without dementia to first examination with dementia) was used.

### Genotype data

Genotyping was performed with the NeuroChip (Illumina) [53]. QC included the removal of participants due to any of the following: per-sample call rate < 98%, sex mismatch, and excessive heterozygosity [FHET (F coefficient estimate for assessing heterozygosity) outside ± 0.2]. Samples were defined as non-European ancestral outliers, and removed, if their first two principal components (PCs) exceeded six standard deviations from the mean values of the European samples in the 1000 Genome global reference population. Closely related samples were removed based on pairwise PI_HAT (i.e., proportion of genome that are in identity-by-descent; calculated using –genome option in PLINK) ≥ 0.2. Further, markers were excluded due to per-single-nucleotide polymorphism (SNP) call rate < 98%, minor allele frequency (MAF) < 0.01, and Hardy–Weinberg disequilibrium (*p* < 1 × 10–6). The Sanger imputation service was used to impute post-QC, using the reference panel of Haplotype Reference Consortium data (HRC1.1) [18, 22]. The variants rs7412 and rs429358 (which define the *ε2*, *ε3*, and *ε4* alleles) in the *APOE* gene were also genotyped with the KASPar^®^ PCR SNP genotyping system (LGC Genomics, Hoddesdon, Herts, UK) or by mini sequencing, as previously described in detail [54].

### Polygenic risk scores and *APOE* genotype

AD-PRSs were generated using stage 1 of the most recent AD GWAS including clinically defined AD phenotypes [55]. SNPs were selected using LD clumping. The European ancestry samples from the 1000-genomes project were used as reference panel to remove variants in LD, all variants 250 kb upstream and downstream of the top signal were removed (*R*^2^ < 0.001). All variants in the *APOE* region (chromosome 19, coordinates hg19: 44,412,079 to 46,412,079) were removed. In this study, we created PRSs including variants that surpassed four *p* value thresholds (*p* < 5e-8, *p* < 1e-5, *p* < 1e-3, *p* < 1e-1), referred to as 5e-8 AD-PRS (including 15 SNPs), 1e-5 AD-PRS (including 57 SNPs), 1e-3 AD-PRS (including 1333 SNPs), and 1e-1 AD-PRS (including 13 942 SNPs). All PRSs were calculated as the sum of the β-coefficient multiplied with the number (or dosage) of effect alleles of each SNP [18, 22]. The AD-PRSs scores were divided into tertiles and participants were categorised as having either low, middle, or high risk. *APOE* genotype was divided into *ε4* carriers *(*ε*4/*ε*2,* ε*4/*ε*3, or* ε*4/* ε*4)* and ε*4* non-carriers *(*ε*2/* ε*2,* ε*3/*ε*3, or* ε*3 /*ε*2).*

### Potential confounders

Information on potential confounders were obtained through semi-structured interviews and health examinations at baseline examinations (1992–93 or 2000–02). Confounders were chosen a priori based on previous literature. Sex, energy intake, birth year, educational level, physical activity level, smoking, body mass index (BMI,), hypertension, diabetes mellitus and serum cholesterol levels were considered potential confounders. Energy intake was measured as kcal/day and BMI as kg/m^2^. Educational level was dichotomized into compulsory primary education (≤ 6 years for birth cohort 1922, ≤ 7 years for birth cohort 1930) versus more than that. Physical activity level was divided into three groups based on a modified Saltin–Grimby physical activity scale [56]: sedentary lifestyle (sedentary/low physical activity level), moderate physical activity level (low to moderate), or high activity level (moderate to high). Hypertension was defined as systolic blood pressure of ≥ 140 mmHg and/or a diastolic blood pressure of ≥ 90 mmHg (yes/no). Smoking was defined as either current smoker or non-smoker (never smoked or previous smoker). Diabetes mellitus was defined as a diagnosis told by a medical doctor, being on antidiabetic drugs, or having a venous blood glucose value of ≥ 11.1 mmol/L (yes/no). In the analyses including gene data, five principal components (PCs) were added as potential confounders to correct for population stratification (differences in allele frequencies due to genetic ancestry).

### Statistical analyses

Chi-square and Mann–Whitney *U* tests (nominal and ordinal variables) and Student’s independent *t* tests (continuous variables) were performed to compare characteristics between participants with and without incident dementia.

The interaction variables *APOE* ε*4* (carrier or non-carrier)*healthy dietary pattern (factor score 1) and *APOE* ε*4* (carrier or non-carrier)*western dietary pattern (factor score 2) were calculated. The interaction variables AD-PRS (low, middle or high risk)*dietary pattern score (factor score 1) and AD-PRS (low, middle or high risk)*dietary pattern score (factor score 2) were calculated for all AD-PRSs (5e-8 AD-PRS*healthy dietary pattern, 1e-5 AD-PRS*healthy dietary pattern, 1e-3 AD-PRS*healthy dietary pattern, 1e-1 AD-PRS* healthy dietary pattern, 5e-8 AD-PRS*western dietary pattern, 1e-5 AD-PRS*western dietary pattern, 1e-3 AD-PRS*western dietary pattern and 1e-1 AD-PRS*western dietary pattern).

Cox regression analyses were performed in two models with the healthy and western dietary pattern scores (factor scores 1 and 2) as independent variables and incident dementia as the dependent variable. In model 1, the analyses were adjusted for sex and birth year. In model 2, the analyses were adjusted for sex, birth year, energy intake, BMI, serum cholesterol, diabetes, hypertension, smoking, education, and physical activity level. Further, Cox regression analyses were performed with the interaction variables as independent variables and incident dementia as the dependent variable. All interaction analyses were performed in two models. In model 1, the analyses were adjusted for sex and birth year. In model 2, the analyses were adjusted for sex, birth year, energy intake, BMI, serum cholesterol, diabetes, hypertension, smoking, education, and physical activity level, and 5 PCs to adjust for population stratification. Similar to other studies, we chose a *p* value threshold of < 0.1 to detect an interaction [26, 28, 29]. To facilitate interpretation of identified interactions and interaction models, effect values for the prediction scores were shown for each genetic group separately.

The time variable in all the Cox regression analyses was calculated as time in years from baseline examination (1992–93 or 2000–02) to either age at dementia diagnosis, age at death or time to end of study (Dec 31, 2016, for those with last examination year 2015–16, and Dec 31, 2012, for those with register data until 2012). Cox model proportional hazard assumptions were verified with Schoenfeld residuals.

The statistical analyses were performed using IBM SPSS STATISTICS 24. R programming versions 3.6.0 were used to test Cox model proportional hazard assumptions.

## Results

Characteristics of the participants who were free from dementia at baseline and had both dietary and genetic data are presented for the total sample and stratified by incident dementia, yes/no in Table 3. During the mean follow-up time of 12.8 years (SD 4.5 years, 7685.5 person-years), 125 participants developed dementia. The mean age of dementia onset was 80.2 years (SD 4.9). There were no significant differences in characteristics between those that developed dementia and those that did not, except for a higher BMI (26 vs. 27) and a higher adherence to the healthy dietary pattern among those that did not develop dementia, and a higher percentage of *APOE ε4* carriers among those that developed dementia (26 vs. 40%).

**Table 3:** Characteristics of participants with dietary and genetic data

	Total (*n* = 602)	Incident dementia (*n* = 125)	No incident dementia (*n* = 477)	*p* values^a^
	Mean (SD) *n* = cases/total case^b^	Mean (SD) *n* = cases/total case^b^	Mean (SD) *n* = cases/total case^b^	
Age at baseline examination	70.6 (0.3) 602/602	70.6 (0.3) 125/125	70.6 (0.3) 477/477	0.49
5e-8 AD-PRS score	− 0.004 (0.958) 602/602	0.013 (0.909) 125/125	− 0.008 (0.972) 477/477	0.83
1e-5 AD-PRS score	− 0.034 (1.020) 602/602	− 0.012 (1.040) 125/125	− 0.039 (1.015) 477/477	0.79
1e-3 AD-PRS score	− 0.042 (1.009) 602/602	− 0.061 (1.124) 125/125	− 0.036 (0.978) 477/477	0.81
1e-1 AD-PRS score	− 0.0182 (1.008) 602/602	− 0.094 (1.027) 125/125	0.002 (1.003) 477/477	0.35
Healthy dietary pattern (factor score 1)	0.075 (1.253) 602/602	− 0.117 (1.315) 125/125	0.125 (1.233) 477/477	0.05
Western dietary pattern (factor score 2)	− 0.039 (1.221) 602/602	− 0.050 (1.249) 125/125	− 0.036 (1.214) 477/477	0.91
Energy intake kcal/day	2182 (636) 602/602	2107 (579) 125/125	2202 (649) 477/477	0.14
BMI	26.8 (4.1) 597/602	26.1 (3.9) 124/125	27.0 (4.2) 473/477	0.03
Serum cholesterol mmol/l	5.9 (1.1) 599/602	6.0 (1.0) 125/125	5.9 (1.1) 474/477	0.45

	% *n* = cases/total case^b^	% *n* = cases/total case^b^	% *n* = cases/total case^b^	
*APOE* ε*4* carriers	29 175/602	40 50/125	26 125/477	0.003
Sex (women)	64 384/602	70 88/125	62 296/477	0.08
Hypertension^c^	80 484/602	77 96/125	81 388/477	0.26
Diabetes mellitus	14 81/602	13 16/125	14 65/477	0.81
Physical activity level				0.93
Sedentary/low	10 59/592	11 13/124	10 46/468	
Low to moderate	46 270/592	44 55/124	46 215/468	
Moderate to high	44 263/592	45 56/124	44 207/468	
Educational level > compulsory^d^	40 238/597	40 50/124	40 188/473	0.91
Current smoker	16 95/595	13 16/124	17 79/471	0.30

^a^*p* values for comparisons between those that developed dementia and those that did not

^b^Total number of cases are presented for all characteristics since there are missing data for some of the variables

^c^Hypertension was defined as systolic blood pressure of ≥ 140 mmHg and/or a diastolic blood pressure of ≥ 90 mmHg

^d^More than 6 years for birth cohort 1922 and more than 7 years for birth cohort 1930

In the total population, we found no associations between adherence to the dietary patterns and incident dementia in either model 1 or model 2 (Table 4).

**Table 4:** Associations between adherence to the dietary patterns and incident dementia in the total sample

Dietary patterns	Model 1^a^			*p* value	Model 2^a^			*p* value
	Dementia *n* = 125/602^b^				Dementia *n* = 121/573^b^			
	HR	95% CI			HR	95% CI		
Healthy (factor 1)	0.87	0.75; 1.02		0.08	0.83	0.67; 1.03		0.09
Western (factor 2)	1.04	0.89; 1.21		0.62	1.06	0.90; 1.25		0.48

HR and 95% CI estimated using a Cox proportional hazards model with follow-up time (years) as the time scale

^a^Model 1 is adjusted for sex and birth year. Model 2 is adjusted for sex, birth year, BMI, energy intake, serum cholesterol level, hypertension, diabetes, physical activity level, educational level, and smoking status

^b^Dementia events/total cases. Those with missing data were excluded in model 2 (*n* = 29 with missing data, *n* = 5 for BMI, *n* = 3 for serum cholesterol, *n*=5 for education, *n* = 10 for physical activity level and *n* = 7 for smoking)

Interactions were observed between adherence to the dietary patterns and *APOE* ε*4* status in relation to incident dementia (interaction *p* value threshold of < 0.1) (Table 5). For the healthy dietary pattern, this interaction was found in model 1, but not in the fully adjusted model 2. For the western dietary pattern, there was an interaction in both model 1 and the fully adjusted model 2 (Table 5).

**Table 5:** Interactions between genetic risk factors and dietary patterns in relation to incident dementia

Interaction models	Model 1^a^		*p *value	Model 2^a^		*p* value
	Dementia *n* = 125/602^b^			Dementia *n* = 121/573^b^		
	HR	CI		HR	CI	
*APOE* ε4*healthy dietary pattern	1.29	0.96; 1.74	0.09	1.13	0.82; 1.54	0.47
Healthy dietary pattern	0.79	0.65; 0.95	0.01	0.77	0.61; 0.98	0.03
*APOE* ε*4* status	1.83	1–27; 2.63	0.001	2.0	1.37; 2.91	0.0003
*APOE* ε4*western dietary pattern	1.31	0.96; 1.79	0.09	1.38	1.01; 1.89	0.05
Western dietary pattern	0.98	0.81; 1.18	0.81	0.99	0.81; 1.21	0.92
*APOE* ε*4* status	1.87	1.31; 2.68	0.001	2.06	1.42; 3.00	0.0001
5e-8 AD-PRS*healthy dietary pattern	0.91	0.77; 1.08	0.29	0.97	0.81; 1.16	0.70
Healthy dietary pattern	1.05	0.72; 1.52	0.81	0.89	0.58; 1.37	0.60
5e-8 AD-PRS	0.98	0.79; 1.22	0.87	0.97	0.77; 1.21	0.77
1e-5 AD-PRS*healthy dietary pattern	0.92	0.78; 1.10	0.36	1.01	0.84; 1.22	0.90
Healthy dietary pattern	1.03	0.70; 1.52	0.88	0.82	0.52; 1.28	0.37
1e-5 AD-PRS	1.09	0.88; 1.36	0.44	1.12	0.89; 1.40	0.35
1e-3 AD-PRS*healthy dietary pattern	1.05	0.88; 1.26	0.59	1.02	0.84; 1.23	0.86
Healthy dietary pattern	0.79	0.54; 1.16	0.23	0.80	0.53; 1.23	0.31
1e-3 AD-PRS	1.05	0.85; 1.30	0.65	1.04	0.84; 1.30	0.70
1e-1 AD-PRS*healthy dietary pattern	0.99	0.82; 1.18	0.88	0.95	0.78; 1.14	0.56
Healthy dietary pattern	0.90	0.61; 1.31	0.57	0.92	0.61; 1.39	0.70
1e-1 AD-PRS	0.94	0.76; 1.17	0.59	0.98	0.78; 1.23	0.88
5e-8 AD-PRS*western dietary pattern	1.10	0.91; 1.32	0.32	1.07	0.89; 1.30	0.47
Western dietary pattern	0.87	0.58; 1.28	0.47	0.93	0.62; 1.39	0.72
5e-8 AD-PRS	1.00	0.81; 1.24	0.99	0.98	0.79; 1.23	0.88
1e-5 AD-PRS*western dietary pattern	1.05	0.87; 1.27	0.62	0.99	0.82; 1.20	0.96
Western dietary pattern	0.95	0.63; 1.42	0.79	1.07	0.71; 1.63	0.74
1e-5 AD-PRS	1.11	0.90; 1.39	0.33	1.12	0.90; 1.41	0.32
1e-3 AD-PRS*western dietary pattern	0.96	0.80; 1.16	0.68	0.98	0.81; 1.18	0.83
Western dietary pattern	1.12	0.76; 1.65	0.57	1.11	0.74; 1.65	0.62
1e-3 AD-PRS	1.05	0.85; 1.30	0.65	1.04	0.83; 1.30	0.72
1e-1 AD-PRS*western dietary pattern	1.06	0.88; 1.28	0.56	1.04	0.85; 1.26	0.71
Western dietary pattern	0.93	0.63; 1.38	0.72	0.99	0.65; 1.50	0.96
1e-1 AD-PRS	0.95	0.77; 1.18	0.65	1.00	0.79; 1.25	0.97

HR and 95% CI estimated using a Cox proportional hazards model with follow-up time (years) as the time scale. *APOE* ε*4* status are divided into carriers/non-carriers. The AD-PRSs are divided into tertiles of low/middle or high risk. Factor score 1 is labelled the “healthy” dietary pattern and factor score 2 is labelled the “western” dietary pattern

^a^Model 1 is adjusted for sex and birth year. Model 2 is adjusted for sex, birth year, BMI, energy intake, serum cholesterol level, hypertension, diabetes, physical activity level, educational level, smoking, and five principal components (PCs) to correct for population stratification

^b^Dementia events/total cases. Those with missing data were excluded in model 2 (*n* = 29 with missing data, *n* = 5 for BMI, *n* = 3 for serum cholesterol, *n* = 5 for education, *n* = 10 for physical activity level and *n *= 7 for smoking)

*APOE* ε*4* non-carriers with a higher adherence to the healthy dietary pattern had a reduced risk of dementia in model 1 (HR 0.79; 95% CI 0.65–0.95) and in the fully adjusted model 2 (HR 0.77; 95% CI 0.61–0.98), but no such associations were found among *APOE* ε*4* carriers (Table 6). There was an association between the western dietary pattern and an increased risk of dementia among *APOE* ε*4* carriers in the fully adjusted model 2 (HR 1.37; 95% CI 1.05–1.78), but not in model 1 (HR 1.28; 95% CI 0.99–1.66). There was no association between the western dietary pattern and an increased risk of dementia among *APOE* ε*4* non*-*carriers (Table 6).

**Table 6:** Associations between adherence to the dietary patterns and incident dementia among *APOE ε4* carriers and *APOE ε4* non-carriers

	Model 1^a^		*p* value	Model 2^a^		*p* value
	Dementia *n* = 125/602^b^			Dementia *n* = 121/573^b^		
	HR	CI		HR	CI	
Healthy dietary pattern						
*APOE* ε*4* non-carriers	0.79	0.65; 0.95	0.01	0.77	0.61; 0.98	0.03
*APOE* ε*4* carriers	1.02	0.80; 1.30	0.89	0.86	0.63; 1.18	0.35
Western dietary pattern						
*APOE* ε*4* non-carriers	0.98	0.81; 1.18	0.81	0.99	0.81; 1.21	0.92
*APOE* ε*4* carriers	1.28	0.99; 1.66	0.06	1.37	1.05; 1.78	0.02

HR and 95% CI estimated using a Cox proportional hazards model with follow-up time (years) as the time scale. To facilitate interpretation of identified interactions between dietary patterns*APOE ε4 in relation to incident dementia, separate effect values for the prediction scores are shown for *APOE* ε*4* carriers and *APOE* ε*4* non-carriers

^a^Model 1 is adjusted for sex and birth year. Model 2 is adjusted for sex, birth year, BMI, energy intake, serum cholesterol level, hypertension, diabetes, physical activity level, educational level, and smoking and five principal components (PCs) to correct for population stratification

^b^Dementia events/total cases. Those with missing data were excluded in model 2 (*n* = 29 with missing data, *n* = 5 for BMI, *n* = 3 for serum cholesterol, *n* = 5 for education, *n* = 10 for physical activity level and *n* = 7 for smoking)

No interactions were found between the AD-PRSs (divided into tertiles of low, middle, or high risk) and the dietary patterns (healthy and western) in relation to incident dementia (Table 5).

## Discussion

We found interactions between dietary patterns and *APOE* ε*4* status in relation to incident dementia among 70-year-olds in a population-based sample followed on average for 13 years. Among *APOE* ε*4* non*-*carriers, we found an association between higher adherence to a healthy dietary pattern and reduced risk of incident dementia. This association was not observed among ε*4* carriers. However, there was an association between higher adherence to a less healthy western dietary pattern and an increased risk of dementia among ε*4* carriers, but not among ε*4* non*-*carriers. There were no interactions between the AD-PRSs and the dietary patterns in relation to incident dementia.

We could not find other studies that investigated interactions between different dietary patterns and both AD-PRS scores and *APOE* ε*4* status in relation to incident dementia. Some studies have, however, investigated interactions between single genetic variants (usually *APOE* ε*4*) and either single foods or nutrients, or dietary patterns, in relation to either dementia or cognitive function [25–27, 30, 31, 57]. The PREDIMED-NAVARRA intervention study (mean age 67) found an interaction between *CLU* and MeDi in relation to cognitive function, but no other gene–MeDi interactions (*CR1, PICALM, APOE*) were found [25]. A study from the Rush Memory and Aging Project found a marginally statistically significant interaction between the MIND (Mediterranean–DASH Intervention for Neurodegenerative Delay) diet and *APOE* ε*4* in relation to AD [23]. Similar to our study, those two studies found higher protective effects of a healthy dietary pattern among those with a favourable genetic predisposition [23, 25]. In our study, we did not find any interactions between the AD-PRSs and the dietary patterns in relation to incident dementia. One previous study has investigated the interplay between AD-PRS and dietary patterns in relation to cognitive function [26]. They found that poorer diet quality interacted with a 12 SNP AD-PRS (including *APOE*) in relation to lower scores on verbal fluency among African Americans (mean age 57) [26]. However, their results are somewhat hard to compare with ours since we used non-*APOE* AD-PRSs, and dementia was the outcome. The Three-city cohort study and the Cardiovascular Health Cognition study found interactions between fish consumption and *APOE* ε*4* in relation to dementia among older adults (age > 65 years), and that consumption of fish was associated with reduced risk among ε*4* non-carriers, but not among ε*4* carriers [27, 57]. The Three-City cohort study also investigated interactions between n-3 fatty acids, a less healthy dietary pattern and *APOE* ε*4* in relation to dementia, but no interactions were found [27]. Another study from the Rush Memory and Aging Project found interactions between n-3 fatty acids, seafood and *APOE* ε*4* in relation to cognitive function among older adults (mean age 84 years), and that consumption of seafood and n-3 fatty acids was associated with slower global cognitive decline among *ε4* carriers, but not among *ε4* non-carriers [30]. An observational study with pooled participants from the Three-City study and 4 US cohorts (Nurses’ Health study, Women’s health study, Chicago Health and Aging project and Rush Memory and Aging projects) found no interactions between fish consumption and *APOE ε4* and 11 other AD-related genes in relation to cognitive decline among older adults (age > 65 years) [31]. Methodological differences between studies in determining genetic risk, cognitive outcome, age, dietary intake, synergistic effects of foods and nutrients, and differences in food choices and consumption levels could be part of the explanation to why results differ between studies [58].

The dietary patterns derived from the RRR analysis in our study resembles dietary patterns previously associated with dementia risk [3, 8, 11]. The healthy dietary pattern loaded high on foods found in dietary patterns that have been associated with reduced risk (i.e., MeDi) [59], and a higher adherence to the healthy pattern correlated positively (strongest of the two dietary patterns) with the potentially risk reducing nutrients included as response variables in the RRR analysis (fibre, folate, vitamin E and C, and polyunsaturated fatty acids) [3, 41–44]. The western pattern loaded high on foods found in dietary patterns associated with increased risk of dementia [8], and a higher adherence correlated positively with saturated fat intake, and negatively with vitamin C, folate, and fibre intake. Both dietary patterns in our study correlated with higher alcohol intake, but the correlation was stronger with the western dietary pattern, indicating that alcohol may be a more detrimental factor in the western pattern [46]. Even though the healthy pattern in our study also contained food groups that could be included in a western pattern (red meat, desserts, and sweet bakery), we did find an association between higher adherence and reduced risk of dementia among APOE ε*4* non-carriers. A reason for that may be that a healthy diet may attenuate the adverse effects of a western diet [13].

Mechanisms underlying potential *APOE*-diet interactions in relation to dementia risk may involve altered metabolism of n-3 fatty acids, glucose or ketones, impaired transport of fatty acids and cholesterol, or modification of other risk factors where *APOE* status is involved [6, 28]. The *APOE* ε*4* allele has been associated with cardiovascular conditions such as hypercholesterolemia, hypertriglyceridemia (lipid metabolism), increased insulin resistance, enhanced response to inflammation (chronic inflammation), and increased atherosclerosis (vascular system) [60], which may provide links between diet and *APOE* ε*4* carriership in relation to dementia risk since several of these cardiovascular conditions also are affected by dietary factors [5, 6, 44, 61, 62]. It might be that the effect of *APOE* ε*4* carriership diminishes the effect of a healthy diet on dementia risk by intervening at specific sites in the pathogenetic process, *e.g.*, related to cholesterol metabolism. It has also been suggested that diets rich in refined carbohydrates could promote dementia and AD through insulin resistance [63], especially among *APOE* ε*4* carriers [64]. A study from the from the French Three-City Study found an interaction between *APOE* ε*4* and high glycemic load (GL) afternoon snacks, and that high afternoon-snack GL was associated with increased dementia and AD risk in *APOE*-ε4 carriers [64]. However, mechanisms remain elusive and there could be other explanations for an interplay between diet and *APOE* ε*4* status in relation to dementia. Protective effects from diet might not compensate the higher genetic risk associated with *APOE* ε*4* carriership, which could explain why we only found risk reducing effects of a healthy dietary pattern among *APOE* ε*4* non*-*carriers. However, *APOE* ε*4* carriers could potentially still be vulnerable for detrimental effects of less healthy dietary patterns [65], as our results suggest.

### Strengths and limitations

This is one of few studies that have investigated interactions between different dietary patterns and both *APOE* ε*4* status and non-*APOE* ε*4* PRSs in relation to dementia. Strengths of this study are the systematically selected population-based sample, the comprehensive examinations and long follow-up time. The neuropsychiatric examinations were performed by psychiatric nurses and the dementia diagnoses were set in accordance with established diagnostic criteria. The diet history method in this study is validated, and the dietary interviews were performed by registered dietitians, which may reduce misreporting of dietary intake (e.g., recall, over–under reporting), common in dietary examinations [35, 66]. Another strength was the use of the RRR analysis to derive dietary patterns, since it considers both a priori knowledge about dietary factors related to dementia and explores dietary patterns that exist in this population.

The outcome in this study was incident dementia, which may include subtypes of dementia that are not as strongly linked to the genetic risk factors as AD. This could potentially have attenuated the results. It was not possible to perform sub-analyses since there were no data available for subtypes of dementia in the 2015–16 examination. However, in Swedish populations, approximately two-thirds of those with dementia have AD [67, 68]. Moreover, the sample size may be too small to detect a potential interaction between the AD-PRSs and diet in relation to dementia since the effect of the AD-PRSs is not as strong as *APOE* ε*4.* We found an interaction between the western dietary pattern and *APOE* ε*4* status in relation to incident dementia in both adjusted models. For the healthy dietary pattern, we found an interaction in the model that was adjusted for sex and birth year, but not in the fully adjusted model. The results should, therefore, be interpreted with caution. Dietary intake may change during life, and since we do not have dietary data before or after the age of 70, we cannot determine potential effects of dietary intake earlier or later in life. Moreover, the analyses were performed on a Swedish population, and the possibility to generalize the results to non-Caucasian populations is limited.

## Conclusions

The results of this study suggest that there is an interplay between *APOE* ε*4* status and adherence to dietary patterns in relation to incident dementia. Higher adherence to a healthy dietary pattern was associated with a reduced risk of dementia among ε*4* non-carriers, but not among ε*4* carriers. A higher adherence to a western dietary pattern was associated with increased risk of dementia among *ε4* carriers, but not among ε*4* non-carriers. This suggests that diet may play a role and that the effects of risk or risk reducing dietary patterns could differ depending on ε*4* carriership*.* These findings could be of importance for precision nutrition in dementia prevention strategies and for future intervention studies investigating the effect of dietary patterns in relation to dementia incidence.

## Supplementary Information

Below is the link to the electronic supplementary material.

Supplementary file1 (DOCX 16 KB)

Supplementary file2 (SAS 2 KB)

## Data Availability

The datasets used and/or analysed during the current study are available from the corresponding author on reasonable request.

## Abbreviations

APOE: Apolipoprotein E
AD: Alzheimer’s disease
PRS: Polygenic risk score
MeDi: Mediterranean diet
MIND: Mediterranean-DASH Intervention for neurodegenerative delay
GWAS: Genome-wide association studies
SNP: Single-nucleotide polymorphism
RRR: Reduced rank regression
PC: Principal component
