# Supplementary material for: Interactions between dietary patterns and genetic factors in relation to incident dementia among 70-year-olds
Source: Eur J Nutr. 2021 Oct 10;61(2):871–84. doi: 10.1007/s00394-021-02688-9 (PMC8854136; doi:10.1007/s00394-021-02688-9)
Supplement: Supplementary file 1 — Supplementary file1 (DOCX 16 KB) [file 394_2021_2688_MOESM1_ESM.docx]

**Supplementary Table 1.** **Food group content**

| 1 | Fish and shellfish/seafood | Fish, seafood, caviar, and spawn, and dishes containing fish and seafood |
| --- | --- | --- |
| 2 | Meat and processed meat | Meat, processed meat, tripe, offal and blood meals and dishes containing meat and processed meat |
| 3 | Poultry | Poultry and poultry dishes |
| 4 | Eggs | Egg and egg dishes |
| 5 | Potatoes | Potatoes and potato dishes |
| 6 | Vegetables and pulses, nuts, and seeds | Vegetables, root vegetables, mushrooms, pulses, and dishes containing vegetables and pulses, nuts, and seeds |
| 7 | Fruits and berries | Fruits and berries including canned or dried |
| 8 | Keyhole milk products* | Keyhole milk products; fat content ≤0.7 % and for flavoured products limit for sugars ≤9% |
| 9 | Non-Keyhole milk products* | Non-Keyhole milk products, including sweet milk drinks |
| 10 | Cream and crème fraîche | Cream, sour cream and crème fraîche |
| 11 | Cheese | Cheese and cheese dishes |
| 12 | Fast food and savoury bakery | Burgers, doner-kebab, falafel, pizza, hot dogs, taco meals, savoury bakery, pastry, savoury pancakes and crêpes, sandwich layer cake, taco shells, crackers, savoury pie |
| 13 | Pasta, rice, and food grain | Pasta, rice, food grains and pasta, rice, and food grain dishes |
| 14 | Bread refined | Bread refined, soft bread and crisp bread ≤5 % fibre |
| 15 | Bread fibre-rich | Bread fibre-rich, soft bread and crisp bread >5 % fibre |
| 16 | Cereals | Breakfast cereals (hot and cold), porridge and gruel, starch oatmeal |
| 17 | Sweet bakery | Buns, cookies, cakes |
| 18 | Desserts | Sweet pie, crumble, chocolate mousse, cheesecake, sweet soups, ice cream, etc. |
| 19 | Sweet condiments | Sugar, syrup, honey and sweeteners, jam, marmalade, sweet cacao powder |
| 20 | Sweets, candy, and chocolate | Sweets, candy, candy bars, chocolate |
| 21 | Soups | Soups, broth |
| 22 | Sauces, dressings, and condiment | Sauces, dressings, aioli, coleslaw, mayonnaise salads, ketchup, HP sauce |
| 23 | Margarine | Table margarine and soft margarine for cooking, including butter-based |
| 24 | Butter | Butter, lard |
| 25 | Vegetable oil | Vegetable oils |
| 26 | Juice | Fruit and vegetable juices including pure fruit shots |
| 27 | Coffee | Coffee |
| 28 | Tea | Tea |
| 29 | Soda | Soda, lemonade, sports and energy drinks, non-alcoholic cider, regular and light |
| 30 | Alcoholic beverages | Wine, beer, alcoholic cider, drinks, spirits, liqueur ≥1 % alcohol |

* Keyhole is the Swedish National Food Agency-labelling scheme, which guides healthy food choices. For milk and yogurt to meet the criteria for the Keyhole, fat content has to be limited to a maximum of 0.7%, and for flavoured products there is an additional limit for sugars with a maximum of 9 %.
